# SINE Insertion in the Intron of Pig *GHR* May Decrease Its Expression by Acting as a Repressor

**DOI:** 10.3390/ani11071871

**Published:** 2021-06-23

**Authors:** Cai Chen, Yao Zheng, Mengli Wang, Eduard Murani, Enrico D’Alessandro, Ali Shoaib Moawad, Xiaoyan Wang, Klaus Wimmers, Chengyi Song

**Affiliations:** 1 College of Animal Science and Technology, Yangzhou University, Yangzhou 225009, China; chencai9596@hotmail.com (C.C.); zhengyao3225@outlook.com (Y.Z.); wangmengli24021@outlook.com (M.W.); Ali.shoeib@agr.kfs.edu.eg (A.S.M.); wxyan@yzu.edu.cn (X.W.); 2 Leibniz Institute for Farm Animal Biology (FBN), 18196 Dummerstorf, Germany; murani@fbn-dummerstorf.de (E.M.); wimmers@fbn-dummerstorf.de (K.W.); 3 Unit of Animal Production, Department of Veterinary Science, University of Messina, 98168 Messina, Italy; edalessandro@unime.it

**Keywords:** pig, *GH/IGF* axis, retrotransposon insertion polymorphism, SINE, structural variation, *GHR*

## Abstract

**Simple Summary:**

*GH/IGF* axis genes play a central role in the regulation of skeletal accretion during development and growth, and thus represent candidate genes for growth traits. Retrotransposon insertion polymorphisms are major contributors to structural variations. They tend to generate large effect mutations resulting in variations in target gene activity and phenotype due to the fact that they carry functional elements, such as enhancers, insulators, or promoters. In the present study, RIPs in four *GH/IGF* axis genes (*GH*, *GHR*, *IGF1*, and *IGF1R*) were investigated by comparative genomics and PCR. Four RIPs in the *GHR* gene and one RIP in the *IGF1* gene were identified. Further analysis revealed that one RIP in the first intron of *GHR* might play a role in the regulation of *GHR* expression by acting as a repressor. These findings contribute to the understanding of the role of RIPs in the genetic variation of *GH/IGF* axis genes and phenotypic variation in pigs.

**Abstract:**

The genetic diversity of the *GH/IGF* axis genes and their association with the variation of gene expression and phenotypic traits, principally represented by SNPs, have been extensively reported. Nevertheless, the impact of retrotransposon insertion polymorphisms (RIPs) on the *GH/IGF* axis gene activity has not been reported. In the present study, bioinformatic prediction and PCR verification were performed to screen RIPs in four *GH/IGF* axis genes (*GH*, *GHR*, *IGF1* and *IGF1R*). In total, five RIPs, including one SINE RIP in intron 3 of *IGF1*, one L1 RIP in intron 7 of *GHR*, and three SINE RIPs in intron 1, intron 5 and intron 9 of *GHR*, were confirmed by PCR, displaying polymorphisms in diverse breeds. Dual luciferase reporter assay revealed that the SINE insertion in intron 1 of *GHR* significantly repressed the *GHR* promoter activity in PK15, Hela, C2C12 and 3T3-L1 cells. Furthermore, qPCR results confirmed that this SINE insertion was associated with a decreased expression of *GHR* in the leg muscle and longissimus dorsi, indicating that it may act as a repressor involved in the regulation of *GHR* expression. In summary, our data revealed that RIPs contribute to the genetic variation of *GH/IGF* axis genes, whereby one SINE RIP in the intron 1 of *GHR* may decrease the expression of *GHR* by acting as a repressor.

## 1. Introduction

Growth hormone (*GH*) and insulin-like growth factor (*IGF*) represent a part of the hypothalamus/pituitary axis that consists of many other regulatory hormones, receptors, binding proteins, and proteases. *GH* plays a central role in controlling circulatory *IGF1* levels, while circulating *IGF1* provides negative feedback to the pituitary and regulates *GH* secretion [1]. The *GH/IGF* axis plays a central role in skeletal growth regulation and mineral acquisition during development and growth [2]. During puberty, signals of the *GH/IGF* axis are upregulated in both humans and mice, and are correlated with changes in bone formation markers [3,4]. *GH* and *IGF1* promote postnatal growth by both independent and common functions [5]. Growth hormone receptor (*GHR*) is a specific transmembrane receptor of growth hormone (*GH*) which plays an important role in the growth and development of animals. In human studies, a mutation in the *GHR* gene results in *GH* insensitivity syndrome (Laron’s syndrome). Laron’s syndrome patients exhibit short stature and reduced serum *IGF1* [6,7]. Likewise, overexpression of a *GH* antagonist will blunt activation of the *GHR*, resulting in a ~45% reduction in total femoral bone mineral content [8]. *IGF1R* is a tyrosine kinase receptor for *IGF1* and regulates cell metabolism, growth, and differentiation in various mammalian tissues [9], and can be activated by *IGF1*. In addition, abnormal *IGF1R* signaling is associated with many disorders, notably diabetes [10] and cancer [11]. Besides growth, altered *GH* and *IGF1* secretion is also associated with the aging of the immune system [12]. Furthermore, alterations of the *GH/IGF1* axis contribute to the pathogenesis of the cardiovascular disease [13], with, for example, low *IGF1* levels leading to increased risk of ischemic heart disease [14] and stroke [15].

Transposable elements (TEs), which can mobilize to new genomic locations, account for nearly half of mammalian genomes, and can be classified into retrotransposons and DNA transposons based on transposition mechanisms. Retrotransposons constitute a major TE component in mammals, represented by short interspersed nuclear elements (SINEs), long interspersed nuclear elements (LINEs), and long terminal repeats (LTRs) [16,17]. In the pig genome, TEs account for about 40%, of which retrotransposons account for more than 90% of the total TEs [18]. Recently, mounting evidence has suggested that retrotransposons contribute to genome architecture evolution and even maintenance of three-dimensional chromatin organization in mammals [19,20,21]. Retrotransposons often contain enhancers, promoters, and other regulatory elements which have contributed to the evolution of gene regulatory networks [22,23]. Many phenotypic changes caused by retrotransposon insertion have been observed in humans and animals. More than 100 cases of retrotransposon-mediated insertions causing human genetic diseases have been confirmed [24]. Also, in farm animals, there are numerous examples, such as an association of a 275-bp SINE insertion into the first intron of *PDIA4* gene with litter size in pigs [25]. Three SINE insertion polymorphisms in the *VRTN* gene have been identified [26]; one of them has been suggested as a potential causative mutation contributing to vertebral number variation in domestic pigs [27,28]. In dogs, a SINE element located in intron 2 of *IGF1* was found in all small dog breeds that is almost entirely absent from large breeds [29]. A SINE inserted in the *SILV* gene was found to be responsible for merle patterning of the domestic dog [30]. ERV insertion causing a change of the eggshell color in chickens has been reported [31]. Two cases of phenotype changes associated with L1 insertions were also reported in pigs [32,33].

Genetic variation (mainly SNPs) of *GH/IGF* and its association with gene activity and phenotypic variation have been reported extensively [34,35,36]. However, the contribution of retrotransposons to the structural variations of *GH/IGF* axis genes has not been explored yet. In the present study, retrotransposon insertion polymorphisms in four *GH/IGF* axis genes (*GH*, *GHR*, *IGF1*, and *IGF1R*), which have been suggested as major candidate genes of animal growth and body size [5,37,38,39], were investigated, along with their breed distribution. Six RIPs from four genes in the *GH/IGF* axis were identified. The genetic effects and molecular function of one RIP were also evaluated, and it was revealed that it might be involved in the regulation of *GHR* expression by acting as a repressor. These findings contribute to understanding the role of RIPs in shaping the genetic and phenotypic variation in pigs.

## 2. Materials and Methods

### 2.1. Sequence Acquisition and Structural Variation Prediction for GH/IGF Axis Genes

The genic sequences of *GH* (ENSSSCG00000034212), *GHR* (ENSSSCG00000016866), *IGF1* (ENSSSCG00000000857), and *IGF1R* (ENSSSCG00000030560) gene and their flanking regions (5-kb 5′ upstream and 3-kb 3′ downstream) from the reference (Duroc) genome (Sscrofa11.1) were downloaded from Ensembl (http://asia.ensembl.org/index.html, accessed on 1 November 2020). Then, 1000 bp upstream and 1000 bp downstream boundary sequences of each genic region were used to Blast the WGS (whole genome shotgun sequence) to define the same genomic positions for the assembled nonreference genomes of 15 breeds (Table S1). These breeds represented different types of pigs, including miniature pigs (Ellegaard Gottingen minipig, Tibetan pig, Wuzhishan, Bama), lean type pigs (Duroc, Hampshire, Cross-bred (Yorkshire × Landrace × Duroc), Berkshire, Pietrain, Landrace, Yorkshire), and fat type pigs (Bamei, Jinhua, Rongchang, Meishan). The genic sequences (*GH*, *GHR*, *IGF1* and *IGF1R* genes and their flanks) were extracted based on the defined genomic coordinates. However, some genes, particularly for large genes (*GHR* and *IGF1R*), distributed in two or more contigs or scaffolds in some breeds, were manually assembled, with gaps remained, to facilitate alignment. The genomic coordinates were listed in Table S2. Finally, the structural variations were visually inspected based on the multiple alignments of these genes by the ClustalX program (version 2.0, default parameters) [40]. Only the large structural variations (>50 bp) were retained for further analysis.

### 2.2. Retrotransposon Annotation and Insertion Polymorphic Prediction

Retrotransposon annotation of *GH*, *GHR*, *IGF1*, and *IGF1R* gene and their flanking sequences were done using RepeatMasker [41] (version-4.0.9, -nolow) with a custom repeat library obtained from a previous study including 1229 sequences (286 DNA transposon, 238 LINEs, 337 LTRs, 40 SINEs and other repeat sequences). Some LINEs and LTRs were divided into two or more fragments, representing different regions of the retrotransposons. The custom library is available as a supplementary material in the published paper [18]. Only sequence segments displaying a cutoff score of more than 1000 and longer than 100 bp for the masking repeats were retained for further analysis, and only the structural variations overlapping at least 50 bp with retrotransposons were designated as retrotransposon insertion polymorphic sites (RIPs).

### 2.3. RIP Verification and Genotyping

Two individuals of 12 domestic pig breeds, i.e., Bama miniature, Wuzhishan, Congjiangxiang, Jiangkouluobo, Tibetan, Meishan, Sujiang, Ningxiang, Daweizi, Duroc, Yorkshire and Landrace pigs were used for insertion polymorphism detection. Sujiang is a new synthetic breed based on crossbreeding Jiangquhai, Fengjing and Duroc pigs. Sushan is another synthetic breed that includes Meishan, Erhualian and Yorkshire genetics. Meishan and Erhualian breeds originate from Jiangsu, Congjiangxiang and Jiangkouluobo from Guizhou, Ningxiang pigs from Hunan, Tibetan, Wuzhishan and Bama pigs from Sichuan, Hainan, Guizhou and Guangxi are Chinese native pigs, respectively. Landrace, Yorkshire and Duroc pigs are international commercial breeds collected from a breeding farm of Anhui Province. Ear tissue was collected in parallel to agricultural procedures (i.e., pulling in ear tags). Total DNA was isolated from ear tissue with MiniBEST Universal Genomic DNA Extraction Kit by following the manufacturer’s instructions (TaKaRa, Dalian, China).

Primer pairs used for RIPs detection were designed based on the flanking sequences of each insertion site in the Sscrofa11.1. PCR amplifications were carried out in a total volume of 20 μL, containing 50 ng of genomic DNA, 2× Taq Master Mix buffer (Vazyme, Nanjing, China), and 10 pmol of each primer. PCR amplification was performed with following cycling conditions: initial denaturation at 95 °C for 5 min, followed by 30 cycles of 95 °C for 30 s, 58 °C for 20 s, and 72 °C for 30 s, and a final extension of 5 min at 72 °C. PCR products were analyzed by electrophoresis on a 1.5% agarose gel in 1× TAE buffer. Gels were stained by ethidium bromide and visualized with UV fluorescence. In addition, the PCR amplification products for transposon insertion and deletion alleles of selected RIPs were further verified by sequencing.

Two commercial breeds (Large White, Landrace), two synthetic breeds (Sujiang and Sushan), and three Chinese native miniature pig breeds (Bama, Mingguang small ear, and Wuzhishan) were used to analyze allele distribution of selected RIPs with sample size 32, 32, 32, 32, 21, 20, 32, respectively. Ear tissues and total DNA were prepared as above. The two commercial breeds are very popular in China, the two synthetic breeds representing the cross between lean type and fat type, were available for us and fit for HWE and Polymorphic information content (PIC) analysis. The genotype and the allele frequencies were calculated, and Hardy-Weinberg equilibrium was tested using the Chi-square test using the Popgene32 software [42]. PIC was calculated according to the formula $\;PIC=1-\sum_{i=1}^{n}p_{i}^{2}-\sum_{i=1}^{n-1}\sum_{j=i+1}^{n}2p_{i}^{2}p_{j}^{2}$. Linkage disequilibrium for the four RIPs in *GHR* genes were performed by Haploview [43].

### 2.4. Dual-Luciferase Reporter Assay

To analyze evolutionary sequence conservation of the *GH*, *GHR*, *IGF1*, and *IGF1R* genes across pig, chacoan-peccary, cow, sheep, dog, horse, human and mouse, the corresponding sequences and annotation information of these genes (Table S3) were retrieved from Ensembl (http://asia.ensembl.org/index.html, accessed on 19 November 2020). The comparative sequence analysis was performed using mVISTA (http://genome.lbl.gov/vista/index.shtml, last accessed on 20 November 2020). The same approach was applied for the conservation analysis of the genomic region (1.5 kb) close to the SINE insertion in the first intron of *GHR* (*GHR*-RIP10). The core promoter region (chr16:27126086-27126971) of *GHR*, was predicted according to the promotor annotation for the human *GHR* gene in Ensembl and a previous study [44], and showed high sequence conservation compared to the human *GHR* core promoter. The putative porcine *GHR* core promoter was amplified from Duroc genomic DNA and cloned into pGL3-basic vectors (Promega, Madison, WI, USA) to form pGL3-GHRpro vectors (primers are listed in Table S4). The pGL3-GHRpro construct was verified by Sanger sequencing. Two SINE RIP alleles (SINE^+/−^) of the *GHR*-RIP10, including short flanking sequences (112 bp upstream and 88 bp downstream), were cloned and sequenced, and subsequently inserted into the pGL3-GHRpro vector for construction of pGL3-GHRpro-SINE^+^ and pGL3-GHRpro-SINE^−^ vectors in sense direction and verified by Sanger sequencing. A total of 2 × 10^4^ HeLa, PK15, C2C12 and 3T3-L1 cells were plated in a 24-well plate and transfected with the constructs by using Transfectamine^TM^ 5000 transfection reagent (AAT Bioquest, Sunnyvale, CA, USA). After 48 h, cells were collected for luciferase activity evaluation by using the dual-luciferase reporter system (Promega, Madison, WI, USA) according to the manufacturer’s protocol with a Modulus™ II Microplate Multimode Reader (Turner Biosystems, Sunnyvale, CA, USA). Three repetitions for each experiment and three independent experiments were performed. HeLa, PK15 and C2C12 cells were cultured in DMEM medium supplemented with 10% fetal bovine serum, 100 U/mL penicillin, and 0.1 mg/mL streptomycin, 3T3-L1 cells were cultured in DMEM medium supplemented with 10% Newborn calf serum, 100 U/mL penicillin, and 0.1 mg/mL streptomycin. All cells were maintained in a humidified atmosphere with 5% CO2 in air at 37 °C and all cell culture reagents were purchased from Thermo Fisher Scientific (Waltham, MA, USA).

### 2.5. Expression Analysis

For expression analyses, 30-day old Sujiang piglets from different families were randomly selected and genotyped for *GHR*-RIP10. Seven female piglets for SINE^+/+^genotype and six female piglets for SINE^+/−^ were selected. Liver, kidney, leg muscle, longissimus dorsi, and back fat were collected after slaughter and quick-frozen by liquid nitrogen and then stored at −80 °C. The mRNA was extracted using standard Trizol methods (Invitrogen, Carlsbad, CA, USA). The first strand of cDNA was prepared according to the manufacturer’s protocol by using FastKing RT Kit (With gDNase, KR116) (TIANGEN, Beijing, China). Then, the mRNA expression of *GHR* was evaluated by quantitative real-time PCR (qPCR) using the qTower3G PCR System (Analytik Jena AG, Thuringia, Germany) in a total volume of 20 μL according to the manufacturer’s instructions (TaKaRa SYBR Premix Ex Taq^TM^, Dalian, China). *GAPDH* was used as an endogenous control. The relative expression level of the gene was calculated by formula 2^−ΔΔCt^ based on the qPCR results. The specificity of the qPCR products was checked on 1.5% ethidium bromide-stained agarose gels and further confirmed using melting curve analyses.

### 2.6. Statistical Analysis

Experimental results were processed by statistical SPSS17.0 software package (SPSS, Inc., Chicago, IL, USA) using T-test, and the data were expressed as mean ± S.D.

## 3. Results

### 3.1. Five RIPs Generated by Retrotransposon Insertions in the Pig GH/IGF Axis Genes

**Figure 1:**
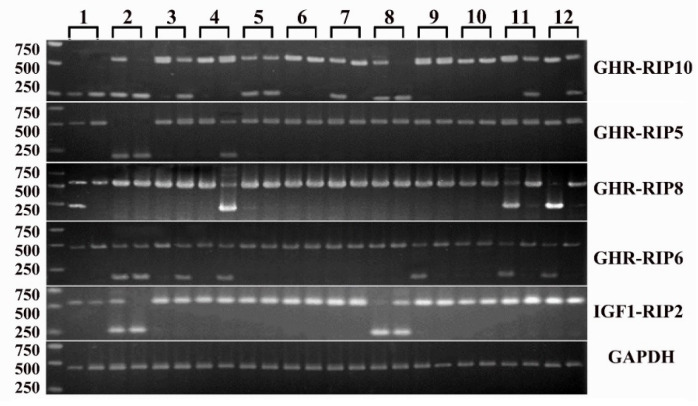
PCR detection of RIPs. 1. Bama miniature, 2. Wuzhishan, 3. Congjiangxiang, 4. Jiangkouluobo, 5. Tibetan, 6. Meishan, 7. Sujiang, 8. Ningxiang, 9. Daweizi, 10. Duroc, 11. Yorkshire, 12. Landrace. M: DNA marker DL2000.

**Figure 2:**
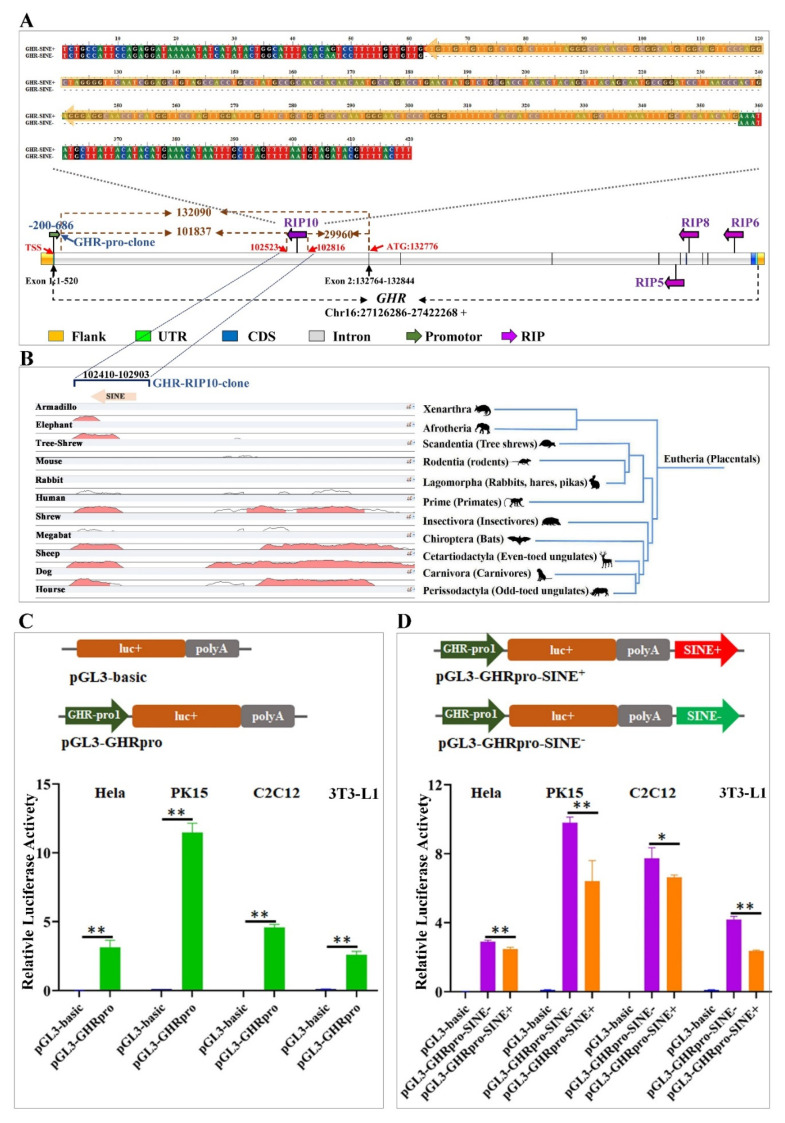
Effect of the GHR-RIP10 SINE insertion on *GHR* promoter activity. (**A**) The SINE sequence in GHR-RIP10 site with and without SINE insertion and its location on *GHR* gene. (**B**) The conservation analysis for the GHR-RIP10 locus. (**C**) *GHR* promotor detection results in Hela, PK15, C2C12 and 3T3-L1 cells by dual-luciferase reporter assay. (**D**) Impact of SINE insertion of GHR-RIP10 on the promoter activity of *GHR* by dual-luciferase reporter assay. * showed *p* < 0.05; ** showed *p* < 0.01.

The sequences of four *GH/IGF* axis genes (*GH*, *GHR*, *IGF1*, *IGF1R*) and their flanking regions (5 kb-5′ upstream and 3 kb-3′ downstream, respectively), which tend to contain most regulatory elements [45], were downloaded or reassembled based on the 16 genome sequences deposited in NCBI as described in the methods section. The reassembly of *GHR* in Tibetan and Goettingen failed due to too many gaps, which resulted in poor alignments with the Sscrofa11.1. The genomic coordinates of the four analyzed *GH/IGF* axis genes and their flanking sequences were summarized in Table S2. Structural variations were identified by multiple alignments using the ClustalX program for each gene. A total of 92 large structural variations (>50 bp) of *GH/IGF* axis genes were obtained and are summarized in Table 1 and Table S5. Out of these, 42 large SVs overlapping with retrotransposon insertions (>50 bp), were designated as putative RIPs (Table 1 and Table S5). Then, these predicted RIPs were investigated by PCR using genomic DNA samples from twelve domesticated pig breeds, which represented different types of pigs, including fat (Meishan, Ningxiang, and Daweizi, Jiangkouluobo), medium (Sujiang), and lean (Duroc, Landrace, and Yorkshire) types, and miniature pigs (Bama, Wuzhishan, Congjiangxiang, Tibetan). Five RIPs were confirmed by PCR with clear polymorphic PCR products across the analyzed DNA samples (Figure 1). In detail, four RIPs in *GHR*, and one RIP in *IGF1* were obtained. Three RIPs in the *GHR* and one RIP in the *IGF1* were generated by SINE insertions (varied from 287 to 301 bp), one RIP in *GHR* was generated by L1 fragment (L1D20) deletion (318 bp). All confirmed RIPs were located in the introns of these genes and designated as the deletion alleles relative to the Sscrofa11.1, four RIPs were oriented in antisense, and one in sense relative to the gene (Table 2). Each RIP was validated by sequencing (Figure 2A and Figure S1).

### 3.2. RIP Distribution in Different Pig Breeds

For the five identified RIPs from *GHR* (GHR-RIP5, GHR-RIP6, GHR-RIP8 and GHR-RIP10) and *IGF*1 (IGF1-RIP2) genes, respectively, their population distribution across the seven breeds was further evaluated by PCR genotyping (Figure S2). These different breeds represent lean type pigs (Landrace, Yorkshire, and Sushan), miniature pigs (Bama, Wuzhishan, Mingguang small-ear), and medium type pigs (Sujiang). The PCR genotyping revealed that three RIPs (GHR-RIP6, GHR-RIP8 and GHR-RIP10) are polymorphic across all seven breeds. The GHR-RIP5 was monomorphic in Sujiang, and the IGF1-RIP1 was monomorphic in Landrace, Yorkshire, and Sujiang respectively, but they segregate in other analyzed breeds (Table 3). For GHR-RIP5, only Large White and Bama were in Hardy-Weinberg equilibrium (*p* > 0.05). But for GHR-RIP6, all breeds were in Hardy-Weinberg equilibrium. For GHR-RIP8 and GHR-RIP10, most breeds (except GHR-RIP8 in Sushan, GHR-RIP10 in Landrace and Bama) were in Hardy-Weinberg equilibrium (*p* > 0.05), and for IGF1-RIP2, Wuzhishan, Bama and Mingguang small ear were in Hardy-Weinberg equilibrium (*p* > 0.05). We did not find obvious linkage disequilibrium for the four RIPs in *GHR* genes (Figure S3). Regarding polymorphic information content (PIC), in most breeds the RIPs display moderate polymorphism with an average PIC of 0.2659 (Table 3).

### 3.3. SINE Insertion in the First Intron of GHR May Repress the Promoter Activity

Comparative sequence analysis using mVista revealed that overall, *GH*, *GHR*, *IGF1*, and *IGF1R* genes are highly conserved across pig, chacoan peccary, cow, sheep, dog, horse, human, and mouse. Furthermore, the RIPs (GHR-RIP5, GHR-RIP6, GHR-RIP8, GHR-RIP9) tend to appear in the downstream introns (intron 5, intron 7 and intron 9) of *GHR* (Table 2), which show relatively lower conservation compared with other regions across these species (Figure S4). However, the 294 bp SINE insertion designated GHR-RIP10 appeared in the first intron of *GHR* in an antisense orientation (Figure 2A and Table 2) and overlaps with a region conserved across mammals (Figure 2B). This resides 101,837 bp far away from the predicted core promoter, 102,523 bp downstream to the TSS, 29,960 bp upstream relative to the translation start site (ATG), 102,003 and 29,948 bp away from to the first exon and second exon, respectively. The SINE insertion was a full length SINE element, which belongs to the SINEA6 subfamily.

To further evaluate the potential roles of the SINE insertion in the regulation of the host *GHR* gene, the core promoter region of *GHR* was cloned into a luciferase reporter vector (pGL3-basic) to form the vector pGL3-GHRpro (Figure 2C), and then subjected to luciferase reporter assay evaluation in HeLa, PK15, C2C12 and 3T3-L1 cell lines. The dual-luciferase reporter assay revealed that the cloned putative core promoter of the porcine *GHR* gene can drive the luciferase expression (Figure 2C). Then, a 494 bp genomic fragment, which contains the 294 bp SINE insertion allele (SINE^+^) and 200 bp genomic flanking regions, or just the flanking sequence (absent SINE insertion allele/SINE^−^), were cloned and inserted downstream of the reporter gene to form the vectors pGL3-GHRpro-SINE^+^ and pGL3-GHRpro-SINE^−^ (Figure 2D), respectively. Then, these vectors were submitted for dual luciferase assay evaluation in HeLa, PK15, C2C12 and 3T3-L1 cell lines. We observed that the construct pGL3-GHRpro-SINE^+^ containing the SINE insertion showed significantly lower promoter activity (*p* < 0.05/*p* < 0.01) compared to the pGL3-GHRpro-SINE^−^ construct in all these four cells (Figure 2D), which suggested that the SINE may act as a repressor in the regulation of *GHR* activity.

### 3.4. Decreased Expression of GHR in Muscle Associated with the SINE Insertion in the First Intron

**Figure 3:**
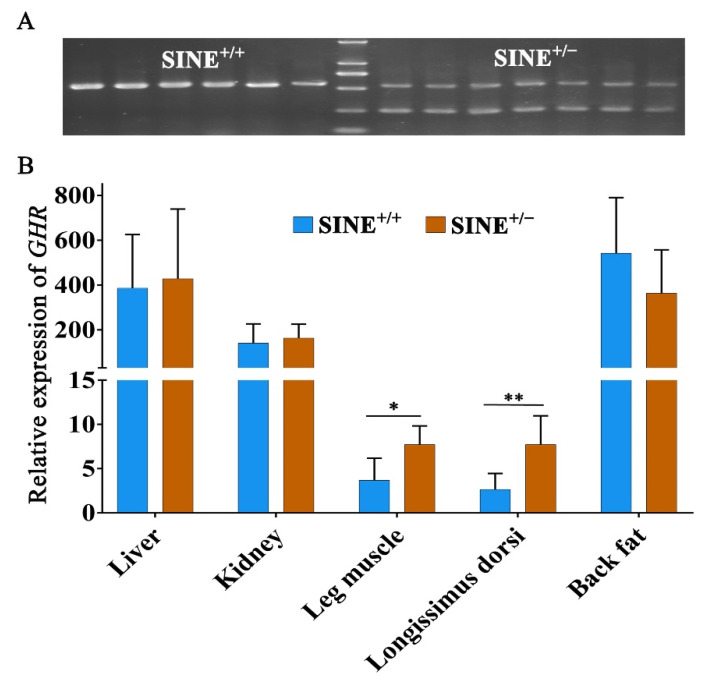
Association of SINE insertion genotype with the expression of *GHR* in tissues of 30-day old piglets. (**A**) Genotype result for 13 Sujiang individuals. (**B**) RT-qPCR results for *GHR* expression. * showed *p* < 0.05; ** showed *p* < 0.01.

To further investigate the functional role of the SINE insertion in the porcine *GHR* gene regulation, we investigated the mRNA expression of *GHR* by qPCR in the liver, kidney, leg muscle, longissimus dorsi, and back fat tissues of 30-day old Sujiang piglets with different genotypes. Firstly, twenty 30-day old Sujiang piglets were genotyped for the *GHR*-RIP10 by PCR. We found only two genotypes in this cohort. For qPCR evaluation 13 individuals, six homozygous for the insertion allele and seven heterozygous, were selected (Figure 3A) and tissues were sampled. The qPCR results revealed that the SINE insertion was associated with lower expressions of *GHR* in the leg muscle and longissimus dorsi. In detail, the expressions of *GHR* in both leg muscle and longissimus dorsi of SINE^+/+^ pigs were significantly lower (*p* < 0.05 or *p* < 0.01) than that in tissues of SINE^+/−^ pigs. The expression level of *GHR* in other tissues (liver, backfat, and kidney) was not significantly different (*p* > 0.05) between the two genotypes (Figure 3B).

## 4. Discussion

Genome-scale studies over the past several decades have shown that TEs and their recognizable remnants, which span both prokaryote and eukaryote organisms, are major determinants of genome sizes [46,47,48]. Retrotransposons occupy one-third to half of mammal genomes [16], while young retrotransposons have been exploited as molecular markers for evolution and population genetic studies in plants [49,50] and humans [51,52]. Most retrotransposons in the pig genome are ancient and no longer transpose, and cannot generate polymorphic insertions in populations, whereas some are young, such as SINEA, the L1D family, and ERV6 subfamilies [18]. These retrotransposons still play roles in shaping genomes and gene evolution and contribute to the genomic variations, and their insertions tend to generate polymorphisms, which can be used as genetic markers. In the present study, we identified four RIPs in the *GHR* gene and one RIP in the *IGF1* gene by mining available pig genome sequences and combining the PCR validation. Four RIPs were generated by SINEA family and one RIP was generated by the L1D family which all belong to the young retrotransposon families in the pig genome according to the previous study [18]. This finding provides additional evidence that the young retrotransposons tend to generate polymorphic insertions, and they still contribute to the genetic and genomic variations in pig. In addition, these RIPs also provide material for the development of molecular markers and proved that there is structural variation in the key genes for growth and development caused by RIPs.

A cross-species sequence comparison for the *GH*, *GHR*, *IGF1*, *IGF1R* genes (shown in Figure S4) showed higher similarity of the porcine sequence with chacoan-peccary, cattle, sheep, dogs and horses, followed by humans, and a lower conservation with mice, which is consistent with the genetic relationship between species. All RIPs confirmed by PCR were located in introns showing a lower level of sequence conservation in these genes. This may be because there is less chance that the retrotransposons inserted into the conserved region will be retained after insertion [53]. Furthermore, our recent study revealed that young SINEs generated a large-scale SINE RIPs occurrence (over 35,000) with an even distribution across chromosomes (14.5 Mb) [54], indicating that SINE may play important roles in genomic variation and evolution in pigs. Here, we found that out of a total of five RIPs confirmed by PCR in the *GHR* and *IGF1* genes, four were derived from SINE insertions, providing additional evidence that SINEs may have an impact on genetic variations in pig. Recently, SINE RIPs, as a new type of genetic markers, were evaluated for population genetic analysis in Chinese miniature pigs, and demonstrated their potential for genetic monitoring and population structure analysis in pigs [55]. Here, our data revealed that the majority of the identified RIPs segregates in diverse breeds with moderate polymorphic information content. Half of them were in Hardy-Weinberg equilibrium in these populations, which thus provide new useful markers for genetic analysis in the pig. The strategy of molecular marker development based on RIPs can be extended to other livestock species since most livestock animals share similar mobilome landscapes [16].

Previous study revealed that about 80% of protein-coding and lncRNA genes contain retrotransposon insertions in pigs [18], and similar annotations were also observed for the bovine, mouse, and human genomes [56,57,58]. Retrotransposons can regulate gene expression by affecting chromatin structure, gene transcription, pre-mRNA processing, or aspects of mRNA metabolism (for a review see [59]). These data suggest that most retrotransposon insertions can alter the activities of nearby genes. During the heat shock response in mouse cells, a small noncoding RNA polymerase III transcript, B2 RNA, which is transcribed from SINE, associates with RNA polymerase II and represses transcription of specific mRNA genes [60,61]. In humans, Alu RNAs can also repress the transcription of some protein-encoding genes upon cell stress [62]. B2 and Alu RNAs potently downregulate transcription by binding to core Pol II with high affinity and specificity, then assemble into preinitiation complexes at the promoter and block RNA synthesis [62,63]. Here, our data demonstrated that the SINE inserted in the first intron of *GHR* is associated with decreased expression of *GHR* in the muscle of Sujiang piglets. In C2C12, 3T3-L1, HeLa and PK15 cell lines, the SINE repressed the activity of the *GHR* promoter in a reporter construct. However, we did not observed apparent alternations of *GHR* expression in fat tissue, liver and kidney between SINE^+/+^ and SINE^+/−^ pigs. This might be due to tissue specificity of the SINE insertion or that the regulation of the gene expression via the insertion is specific to developmental stage. Together, these results suggest that the *GHR*-RIP10 SINE insertion may influence *GHR* gene activity and consequently cause phenotypic variation, as a causative genetic variation. Considering *GHR* as a key gene involved in growth, lean and fat deposition in animals [64], further evaluation of the impact of the GHR-RIP10 on genetic and phenotypic variation in pig is warranted.

## Figures and Tables

**Table 1:** Large structural variations (SVs) predicted by alignment in *GH*, *GHR*, *IGF1*, and *IGF1R* genes and their flanking regions.

	No. of Large SVs				
	Total	*GHR*	*GH*	*IGF1*	*IGF1R*
SVs ≥ 51 bp	92	47	3	7	35
Predicted RIPs	42	26	0	3	13
Confirmed RIPs	5	4	0	1	0

**Table 2:** Genomic information of the identified RIPs by PCR in *GHR* and *IGF1*.

RIP Name	Mutation Type	Chr	Begin	End	TE Type	Orientation Relative to Gene	Length	Gene Structure
GHR-RIP10	Deletion	Chr16	27228809	27229102	SINEA6	Antisense	294	Intron 1
GHR-RIP5	Deletion	Chr16	27388037	27388337	SINEA1	Antisense	301	Intron 5
GHR-RIP8	Deletion	Chr16	27393631	27393854	L1D20	Antisense	318	Intron 7
GHR-RIP6	Deletion	Chr16	27412697	27412983	SINEA1	Antisense	287	Intron 9
IGF1-RIP2	Deletion	Chr5	81790669	81790888	SINEA1	Sense	300	Intron 3

**Table 3:** RIP distribution in different pig breeds.

RIP Name	Polymorphic Breeds	Population Size	Genotype Frequency			Allele Frequency		Hardy-Weinberg Equilibrium Test/*p* Value	PIC
			+/+	+/−	−/−	+	−		
GHR-RIP5	Wuzhishan	32	0.79	0.15	0.06	0.86	0.14	0.0455	0.2118
	Landrace	32	0.00	1.00	0.00	0.50	0.50	0.0000	0.3750
	Large White	32	0.84	0.16	0.00	0.92	0.08	0.6317	0.1364
	Sujiang	32	1.00	0.00	0.00	1.00	0.00	NA	0.0000
	Sushan	32	0.00	1.00	0.00	0.50	0.50	0.0000	0.3750
	Bama	21	0.57	0.43	0.00	0.79	0.21	0.2114	0.2768
	Mingguang small ear	20	0.75	0.15	0.10	0.83	0.17	0.0269	0.2424
GHR-RIP6	Wuzhishan	32	0.00	1.00	0.00	0.50	0.50	0.0000	0.3750
	Landrace	32	0.00	0.81	0.19	0.40	0.60	0.0001	0.3648
	Large White	32	0.00	0.94	0.06	0.47	0.53	0.0000	0.3741
	Sujiang	32	0.00	1.00	0.00	0.50	0.50	0.0000	0.3750
	Sushan	32	0.00	1.00	0.00	0.50	0.50	0.0000	0.3750
	Bama	21	0.00	1.00	0.00	0.50	0.50	0.0000	0.3750
	Mingguang small ear	20	0.00	0.95	0.05	0.48	0.52	0.0001	0.3746
GHR-RIP8	Wuzhishan	32	0.56	0.41	0.03	0.77	0.23	0.4553	0.2915
	Landrace	32	0.03	0.41	0.56	0.23	0.77	0.4553	0.2915
	Large White	32	0.28	0.53	0.19	0.55	0.45	0.6841	0.3725
	Sujiang	32	0.81	0.19	0.00	0.91	0.09	0.5584	0.1504
	Sushan	32	0.19	0.75	0.06	0.56	0.44	0.0030	0.3714
	Bama	21	0.95	0.05	0.00	0.98	0.02	0.9110	0.0384
	Mingguang small ear	20	0.85	0.15	0.00	0.92	0.08	0.3362	0.1364
GHR-RIP10	Wuzhishan	32	0.19	0.44	0.37	0.41	0.59	0.5984	0.3668
	Landrace	32	0.00	0.19	0.81	0.09	0.91	0.0000	0.1504
	Large White	32	0.34	0.53	0.13	0.61	0.39	0.5121	0.3626
	Sujiang	32	0.47	0.47	0.06	0.70	0.30	0.4872	0.3318
	Sushan	32	0.44	0.44	0.12	0.66	0.34	0.3917	0.3481
	Bama	21	0.00	0.76	0.24	0.38	0.62	0.0048	0.3602
	Mingguang small ear	20	0.65	0.35	0.00	0.83	0.17	0.3428	0.2424
IGF1-RIP2	Wuzhishan	32	0.13	0.53	0.34	0.39	0.61	0.5121	0.3626
	Landrace	32	1.00	0.00	0.00	1.00	0.00	NA	0.0000
	Large White	32	1.00	0.00	0.00	1.00	0.00	NA	0.0000
	Sujiang	32	1.00	0.00	0.00	1.00	0.00	NA	0.0000
	Sushan	32	0.00	1.00	0.00	0.50	0.50	0.0000	0.3750
	Bama	21	0.48	0.43	0.09	0.69	0.31	0.9903	0.3363
	Mingguang small ear	20	0.75	0.25	0.00	0.88	0.12	0.5229	0.1889

## Data Availability

All data needed to evaluate the conclusions in this paper are present either in the main text or the Supplementary Materials.

## Supplementary Materials

The following are available online at https://www.mdpi.com/article/10.3390/ani11071871/s1, Figure S1. Sequencing results for PCR amplification of RIPs. Figure S2: PCR detection results of all five RIPs. Figure S3. Linkage disequilibrium for five RIPs in *GHR* gene (indicated by r^2^ × 100). Figure S4. Sequence conservation analysis of pig *GH/IGF* axis genes and their flanking regions across chacoan, cattle, sheep, horse, dog, human and mice, RIPs: Purple arrow, flanking regions: Yellow, CDS: Blue, UTR: green. Table S1. The information of genomes used in this study. Table S2. Assemble information for the *GH, GHR, IGF1* and *IGF1R* genes from the 16 pig genomes. Table S3. The information of sequences used for conservation analysis for the *GH, GHR, IGF1* and *IGF1R* genes. Table S4. Primers for RIPs detection and *GHR* promotor and GHR-RIP10 clone. Table S5. The detail information of structure variations in *GH, GHR, IGF1* and *IGF1R* genes. Supplementary file 1. The sequences of *GH, GHR, IGF1* and *IGF1R* genes from the pig genomes in fasta format.
