# Supplementary material for: SINE Insertion in the Intron of Pig GHR May Decrease Its Expression by Acting as a Repressor
Source: Animals (Basel). 2021 Jun 23;11(7):1871. doi: 10.3390/ani11071871 (PMC8300111; doi:10.3390/ani11071871)
Supplement: Supplementary file 1 [file animals-11-01871-s001.zip › Figure S1-S3-S4-2021-6-6.pdf]

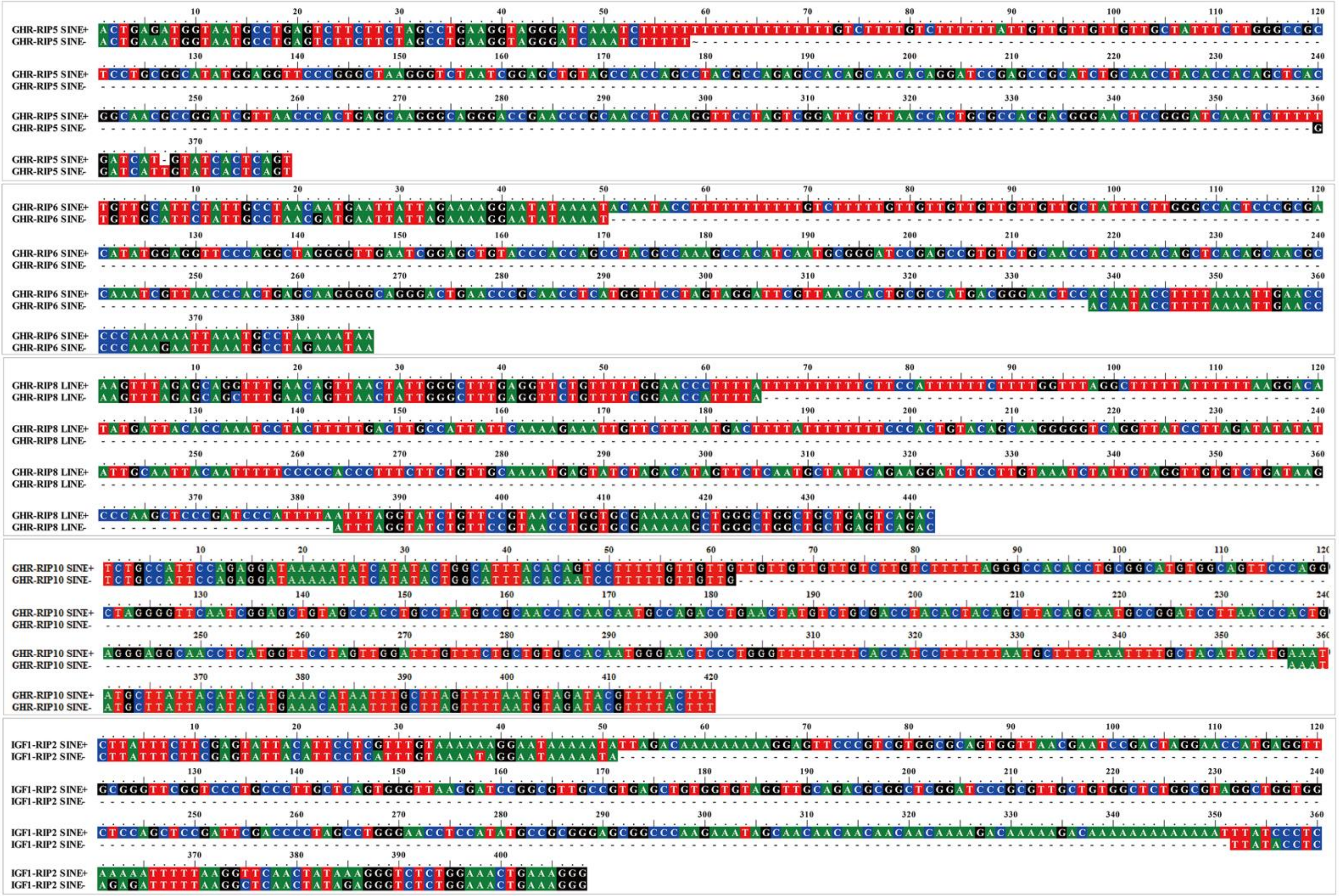

Figure S1. Sequencing results for PCR amplification of RIPs.

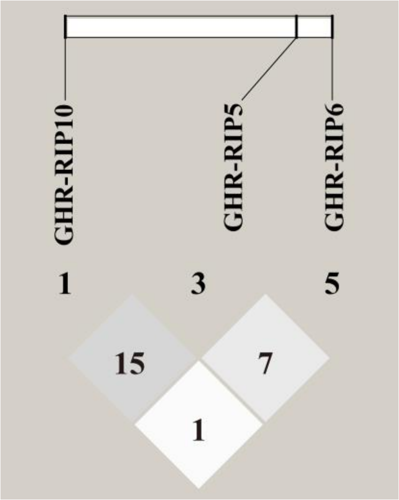

Figure S3. Linkage disequilibrium for five RIPs in *GHR* gene (indicated by $r^2 \times 100$).

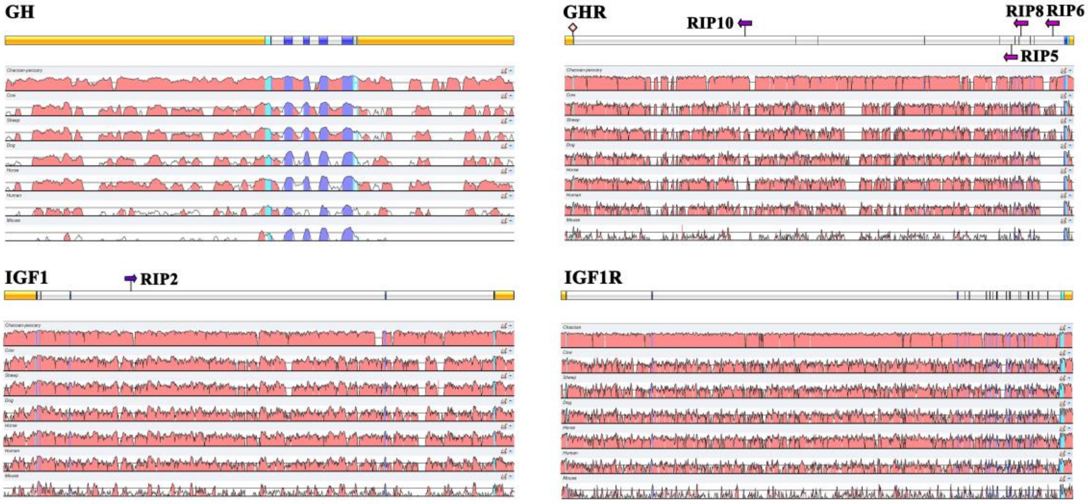

Figure S4. Sequence conservation analysis of pig *GH*/*IGF* axis genes and their flanking regions across chacoan, cattle, sheep, horse, dog, human and mice, RIPs: Purple arrow, flanking regions: Yellow, CDS: Blue, UTR: green
