# Supplementary figures and images for: SINE Insertion in the Intron of Pig GHR May Decrease Its Expression by Acting as a Repressor
Source: Animals (Basel). 2021 Jun 23;11(7):1871. doi: 10.3390/ani11071871 (PMC8300111; doi:10.3390/ani11071871)

**Wuzhishan**

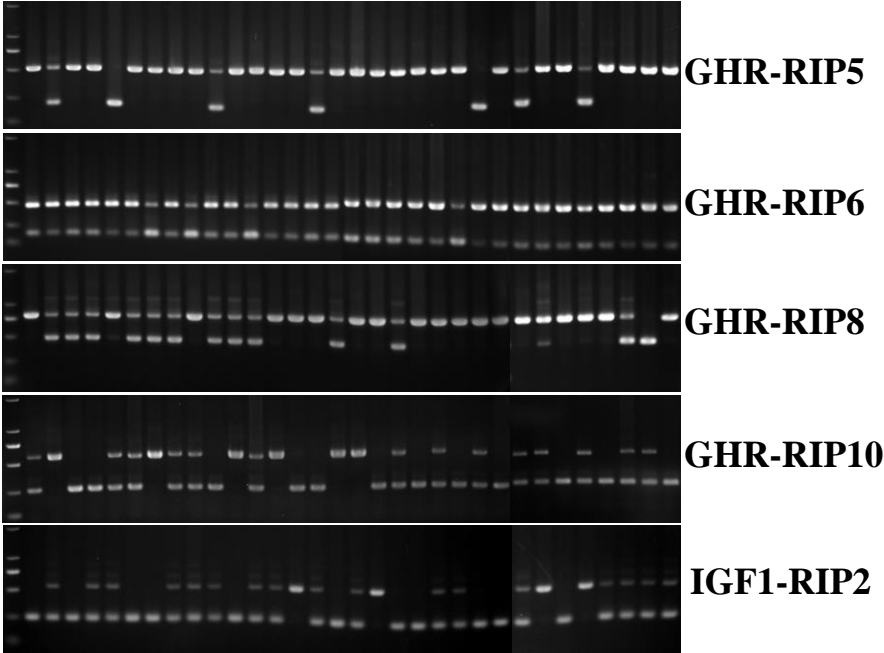

**Landrace**

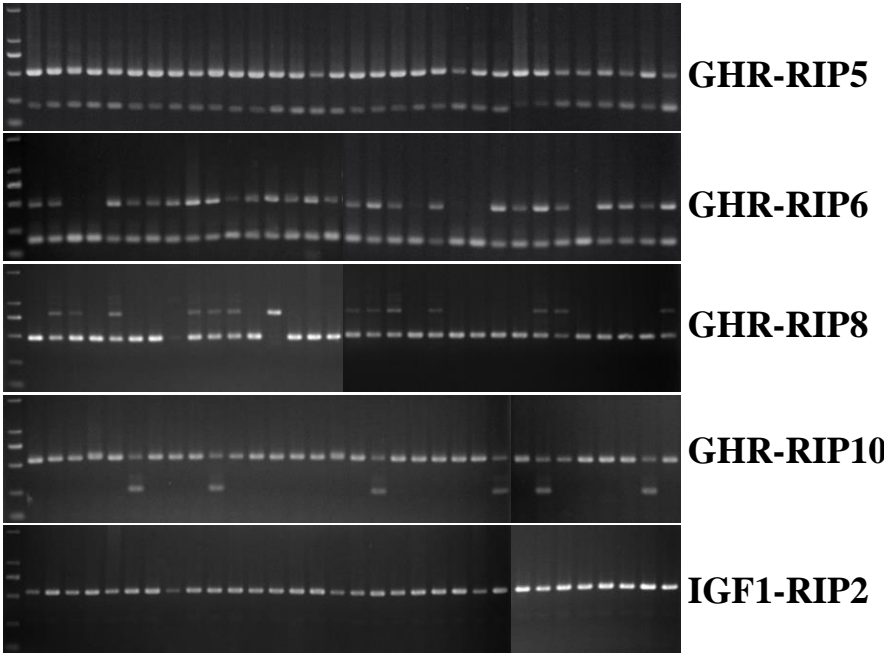

**Sujiang**

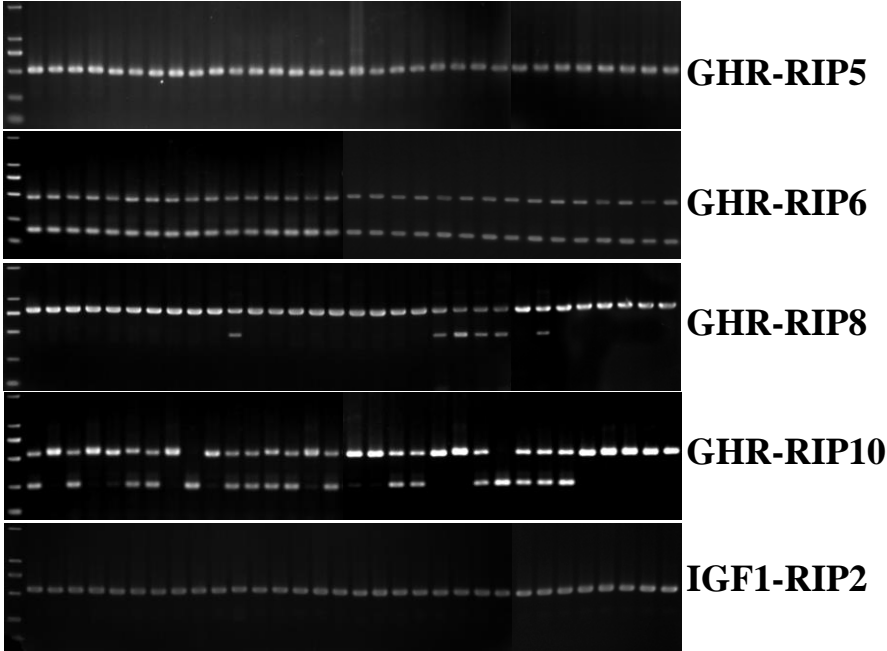

**Sushan**

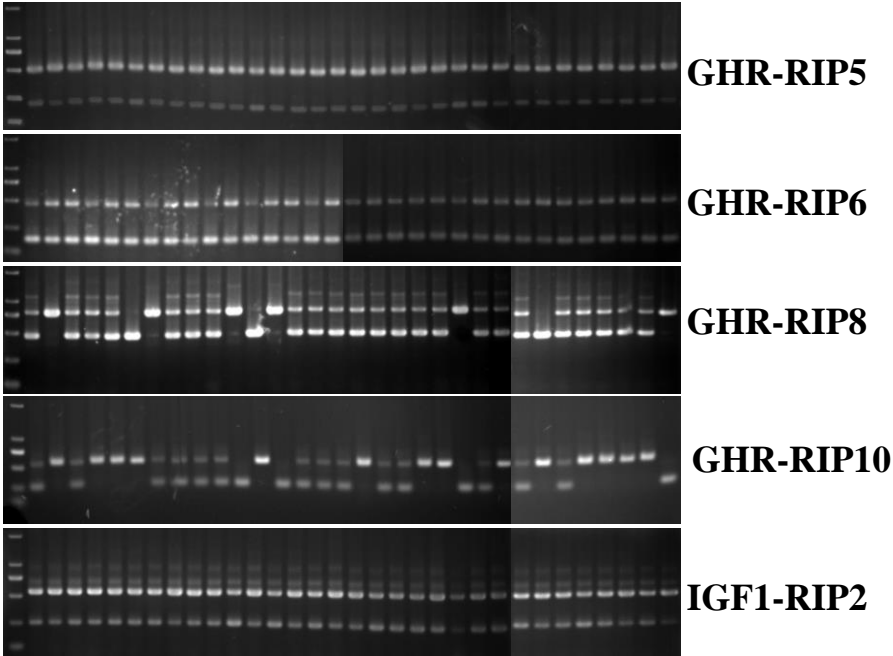

## Yorkshire

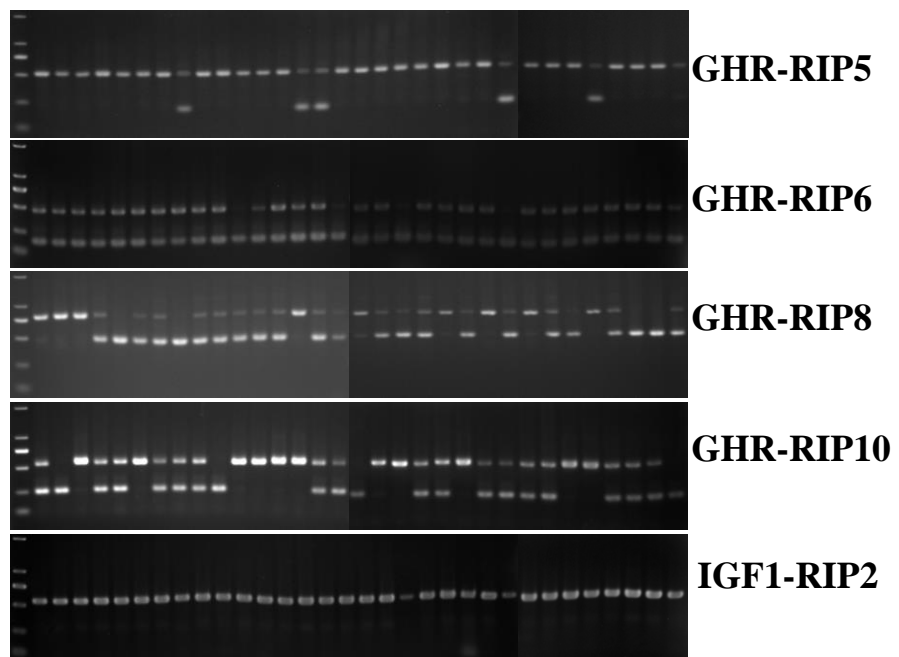

## Mingguang small ear

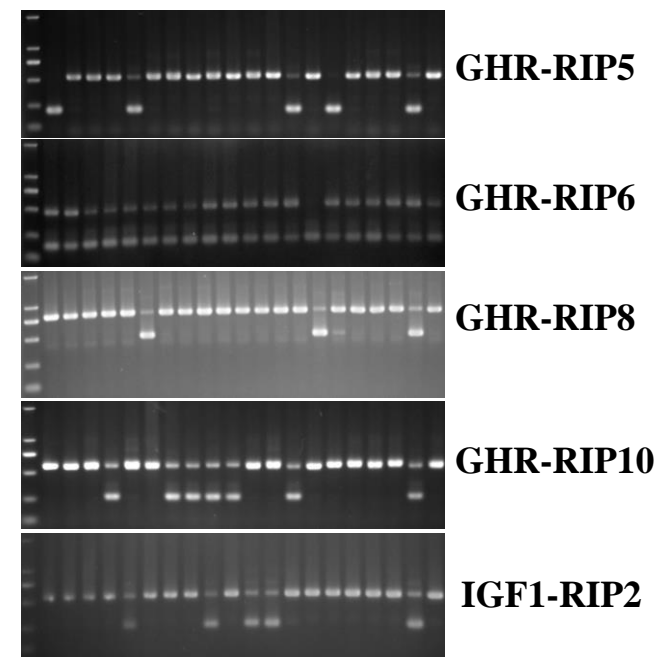

## Bama

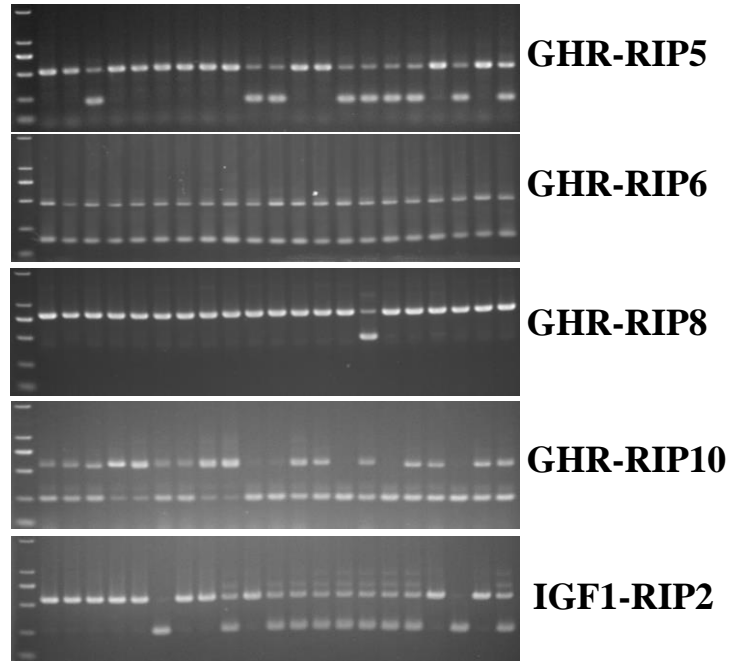

Supplement: Supplementary file 1 [file animals-11-01871-s001.zip › Figure S2-PCR detection results of all five RIPs--2021-6-6.pdf]
